# Entropy, Statistical Evidence, and Scientific Inference: Evidence Functions in Theory and Applications

**DOI:** 10.3390/e24091273

**Published:** 2022-09-09

**Authors:** Mark L. Taper, José Miguel Ponciano, Brian Dennis

**Affiliations:** 1 Department of Ecology, Montana State University, Bozeman, MT 59717, USA; 2 Biology Department, University of Florida, Gainesville, FL 32611, USA; 3 Mathematics Department, University of Florida, Gainesville, FL 32611, USA; 4 Department of Mathematics and Statistical Science, University of Idaho, Moscow, ID 83844, USA; 5 Department of Fish and Wildlife Sciences, University of Idaho, Moscow, ID 83844, USA

**Scope and Goals of the Special Issue:** There is a growing realization that despite being the essential tool of modern data-based scientific discovery and model testing, statistics has major problems. The statistician Ionides speculated that most reported statistical results are false. The perceived failure of scientific studies to reproduce “statistically significant” results has scientists and statisticians alike wringing their hands and despairing of what has been named the “replication crisis”. Public confidence in science is weakening, and perhaps publicity about the replication crisis is contributing to this. Why have these problems in statistics remained uncorrected for so long? There has been a struggle of Wagnerian or titanic proportions between two schools of thought: classical statistics and Bayesian statistics. Neither has prevailed because each school has virtues, and each school has flaws. The virtues impel each school’s adherents to defend, while the flaws give both schools ample opportunity to attack the other. This impasse has been dubbed by the philosopher Deborah Mayo [1] the “Statistics Wars”. In our view, the way forward, the path between the paradigms, is evidential statistics. For many years, there has been a growing understanding of the importance of theory in Ecology and Evolution, with theory being an organized complex of models [2,3,4,5]. Thus, a more model-centric approach to statistics seems appropriate. Practically, science functions by comparing the relative fit of models to data. Science advances when existing (possibly good) models are replaced with new (hopefully better) models. Modern statistical evidence compares the relative support in scientific data for mathematical models. The fundamental tool of model comparison is the evidence function. 

In all of the relevant literatures (philosophical, statistical, and scientific) there is frequent equivocation between the terms (and concepts) of “model” and “hypothesis”. In our usage, a model is a mechanism to generate data or pseudo data. A model may be mathematical, algorithmic, or even physical. A hypothesis, on the other hand, is a statement that may be either true or false. Philosophers, statisticians, and scientists often use the two terms interchangeably. We find this confusing. This is not to say that individual writers are are necessarily confused. For example, the statistician Richard Royal in his ground breaking 1997 volume [6] is quite clear when he says on page 4 that “A simple statistical hypothesis is one that complexly specifies the probability distribution of an observable random variable.” He is describing what we would speak of as a model. However, for every model, there a host of associated hypotheses such as: the model is true, the model describes reality within a specified level of accuracy, the best value for a particular parameter fall within a specified interval, and so forth. 

Royall [7] has said “Statistics today is in a conceptual and theoretical mess.” He noted [7,8] that there are three generic questions regarding models or hypotheses after data have been collected:

1. What do I believe, now that I have this observation?
2. What should I do, now that I have this observation?
3. What does this observation tell me about A versus B? (How should I interpret this observation as evidence regarding A versus B?) (Royall, 1997 page 4).

Royall [6] identified the crux of problem in statistics as a failure of both the classical and Bayesian schools to address head-on a question fundamental to science: “when does a given set of observations support one statistical hypothesis over another?” In his 1997 monograph [6], Royall sought to take a stream of thought running from Venn [9] to Barnard [8] to Birnbaum [10] to Hacking [11] and finally to Edwards [12] elevate it to a river of a paradigm. Royall [6] consciously tried to devise a system that retained virtues of the contesting schools while jettisoning flaws. Royall axiomatically based his evidential statistics on the law of likelihood [11] and the likelihood principle (LP) [10] and utilized the likelihood ratio (LR) as the canonical measure of statistical evidence. This “leads to statistical methods that have a compelling rationale and elegant simplicity” [6].

Unfortunately, the likelihood principle has been strongly critiqued by many. These criticisms fall roughly into three categories: (1) Questions about the legitimacy of Birnbaum’s 1962 derivation (e.g., Mayo [13], but see Gardenberger [14]). (2) Examples where application of the LP leads to paradoxical results (e.g., Cornfield [15]). Additionally, (3) questions about the LP applicability to scientific inference [13,16,17,18,19], given that it was formulated and proved under a perfect model specification scenario, a criterion that is rarely met, if at all, in day-to-day scientific modeling settings.

The greatest problem with the LP is that it is an incredibly limited statement: It only says that if the generating process is exactly given by a known parametric model, then all the information for comparing parameter values is contained in their likelihoods. The principle does not address many things necessary for the prosecution of science. The LP is silent regarding the comparison of different model forms, it is silent as to what happens when the model is misspecified, and it is silent on the quantification of error. Consequently, the LP has been challenged as a foundational tool for scientific inference ever since it was named [20]:

*In advocating the likelihood principle as a basic proposition in the theory of inference, it is necessary to emphasize that it is not proposed that it should be used uncritically unless the model is known very precisely. […] If the model has to be guessed, then the nature of the inference becomes much less precise than is suggested by the formal statement of the likelihood principle.*

(Page 323, Barnard et al. 1962)

We argue that, because scientists are not omniscient, the model virtually always must be “guessed” to some degree. Consequently, the most useful way to think about the LP is as an instructive limiting case—an ideal gas law of sorts. It gives us ideas as to how to fruitfully pursue inference in more realistic cases.

The LR works well as an evidence measure when comparing two statistical models with no unknown parameters under the correct model specification. Such models are known in statistics as “simple hypotheses.” Difficulties arise when model misspecification, model selection, and unknown parameters (including “nuisance parameters”) are involved. In 2004 the statistician Subhash Lele [21] recognized that the log of the likelihood ratio, logLR, between two simple models is an estimate of the contrast of Kullback–Leibler divergences of each of the two models to the underlying generating process. From this realization, he developed the concept of evidence functions as a generalization of the logLR. Evidence functions are essentially contrasts of generalized entropy discrepancies. Corrections can often be constructed, substituting measures that look like the LR or logLR, but are not based entirely on pure likelihood ratios [22,23,24,25]. 

We feel that evidence functions have intuitive appeal and compelling rationale—exceeding even that of the LR. Statistical evidence for one model over another model is just a data-based estimate of the difference in discrepancies of each of two models to the unknown underlying generative process. Furthermore, it is suggested that evidence functions should be developed in accordance with a list of desiderata, each chosen to confer good inferential properties to evidence [21,26,27]. We feel that before proceeding to questions 1 and/or 2, an analyst should endeavor to first answer the evidence question, that is to “let the data speak for themselves” (This quote is often cited as stemming from R. J. Fisher, but we have not been able to verify the attribution). The desiderata in Box 1 are constructed to promote this goal in as clean a statistical fashion through the construction of sound evidence functions.

Box 1 **Desiderata for evidence quoted, with minor emendations, from (Taper and Ponciano [26]).**

D1. Evidence should be a data-based estimate of a contrast of the divergences of each of two models from the data generating process.
D2. Evidence should be a continuous function of data. This means that there is no threshold that must be passed before something is counted as evidence.
D3. The reliability of evidential statements should be quantifiable.
D4. Evidence should be public not private or personal.
D5. Evidence should be portable, that is it should be transferable from person to person.
D6. Evidence should be accumulable: If two data sets relate the same pair of models, then the evidence should be combinable in some fashion, and any evidence collected should bear on any future inferences regarding the models in question.
D7. Evidence should not depend on the personal idiosyncrasies of model formulation. By this we mean that evidence functions should be both scale and transformation invariant.
D8. Consistency, evidence for the true model/parameter is maximized at the true value only if the true model is in the model set, or at the best projection into the model set if it is not.

Using data, evidence functions *estimate* the relative divergence between models and the generating process in some specific metric. Thus, evidence functions can be defined using divergences other than the KL to deal with specific kinds of models, data, or types of questions being asked [21,28]. For some of these cases, such as the important question of choosing between parametric model families, the likelihood principle cannot hold by definition, at least not without modification such as computing differences of expected log-likelihood as suggested by Akaike [29].

This generalization of the LR to evidence functions does not represent any loss of capacity as the LR and all of the substitute measures mentioned above can now be seen as special cases of evidence functions. The log likelihood ratio still holds a place of pride. Lele [21] observed that evidence functions based on other divergences have a lower probability of correct model identifications at every given sample size—under correct model specification. 

Royall [6] made at least two critical advances: First, was the elimination of the null model/alternative model distinction, conceptually and statistically. Both models are treated equally and symmetrically. Additionally, second, the accept/reject dichotomy is replaced with a trichotomy. Possible outcomes are strong evidence for model 1, weak and inconclusive evidence, and strong evidence for model 2. Taper et al. [19] suggest further dividing the outcome continuum into strong evidence for model 1, prognostic evidence for model 1, weak evidence not really favoring either model, prognostic evidence for model 2, and strong evidence for model 2. Prognostic evidence is suggestive of a model, but not sufficiently strong to stand on its own without collateral evidence.

Since then, modern evidential statistics has extended the concept of evidence to statistical cases with unknown parameters and misspecified models, but it realizes that a frequentist error structure can be created for both pre-data design and post-data inference [15,19,23,30]. Further, both conditional uncertainty (i.e., uncertainty in evidence given the data in hand) and unconditional uncertainty (i.e., uncertainty in evidence over resampling of data) can be calculated [19,31].

Testing in classical statistics only conceives of the strength of evidence against a null hypothesis as an error probability, either significance level or size of test. There are several substantial improvements in the handling of errors in evidential statistics from that in classical statistics. First, the concepts of evidence and frequentist error are now distinct, and their inferential impacts can be considered separately [30,32,33]. Second, the asymptotic error structures for evidence, briefly explained below, are vastly superior to those found in Neyman–Pearson hypothesis testing, NPHT. 

It is certainly desirable that inferences should get better as the sample size, *n*, increases. This is not the case for NPHT—even under correct model specification. Inferences are bound to error rates, and Type I error rates (*α*) are constant over all sample sizes. In evidence, the error rates are the probabilities: (a) of misleading evidence, M_i_, and (b) of weak evidence, W. The quantities M_1_ and M_2_ for two models = are roughly analogous to the Type I and Type II error rates *α* and *β* in NPHT. In an evidential analysis, under correct model specification, both M_1_ and M_2_, along with W, go to zero as sample size increases, in strong contrast to the error structure of NPHT. Further, the evidential approach allows an experimenter to control the error by setting a priori the level of evidence, *k*, that will be considered strong [6,23]. Presetting *k* is analogous to setting *α* before conducting an experiment to be analyzed using NPHT.

Model misspecification is deleterious to the error control of both NPHT and evidential model comparisons, but the impacts are much worse for NPHT than for evidence. NPHT realized error rates can be less than, equal to or greater than the nominal size, α, depending on the nature of misspecification, which is disastrous for a procedure that relies entirely on its nominal error rate for inference. To make things even worse, in some realistic situations, realized error rates will actually increase with increasing sample size—eventually reaching a certainty of error [30]. Interestingly, the geometry of the model space is estimable [34] and influences the model identification error probabilities [30]. Further, if nonparametric estimates of data entropy are available then absolute distances from the generating process to models are calculable, not just relative distances [34]. See [19,30,34] for detailed discussions of the impact of model misspecification on evidential assessment.

Under misspecification, the maximum error rates for evidential model identification can be as large as 50%. This occurs when two distinct models are equally divergent from the generating process, an intuitive result. However, for all model comparisons except for this limiting case, both evidential error rates (M and W) decline to zero as sample size increases. 

Another interesting tool for assessing uncertainty in an evidential analysis is the confidence interval for your evidence. Evidence is for comparing models; your evidence is a point estimate of the differences of the divergences each model from the true data generating process. *Even when models are mis-specified*, interval estimates for evidence with valid coverage properties can be produced whenever bootstrapping is possible [19]. 

Thus far, we have compared the evidential approach to statistics with the classical approach, but what about Bayesianism? Bayesian statistical thinking is not a monolith, but a complex stew of shading opinions. I. J. Good [35] waggishly argued that there are at least 46,657 different kinds of potential Bayesians. In seeking some principle common to all he said, “All Bayesians, as I understand the term, believe that it is usually meaningful to talk about the probability of a hypothesis”. It follows from this characterization that Bayesians fundamentally do not address the evidence question because the evidence question is always comparative, see Royall’s question 3 above. For extensive discussion of the confirmation/evidence distinction see [36,37]. While seeking to confirm one’s beliefs is a natural tendency, it runs the risk of being smacked in the head by reality [8]:

*The advantages of odds are even more striking in relation to hypotheses. To speak of the probability of a hypothesis implies the possibility of an exhaustive enumeration of all possible hypotheses, which implies a degree of rigidity foreign to the true scientific spirit. We should always admit the possibility that our experimental results may be best accounted for by a hypothesis which never entered our own heads.*

(Barnard, 1949 page 136)

We can broadly categorize Bayesians into three classes: subjective Bayesians, objective Bayesians, and empirical Bayesians. Further we feel that the empirical Bayes is substantively different from the other two and only Bayes by a historical fluke of nomenclature, as the method could have been just as accurately described as a mixture model or semiparametric likelihoodism if the prior is estimated nonparametrically. 

The distinction between empirical Bayes on one hand and subjective and objective Bayes on the other hand is that the results of an empirical Bayes analysis are invariant to the transformation of the parameters while analyses using subjective or objective Bayes are not. This lack of invariance is not just a philosophical problem but has real world consequences [38].

There is a tendency among scientists to use “noninformative priors”, perhaps because personal idiosyncrasy of subjective priors seems somehow inappropriate for science. We applaud this impulse but point out that the solution is apocryphal. A corollary to the problem of transformation dependence in Bayesian analysis is that noninformative priors do not really exist except for categorical models. Every noninformative prior is informative for another transformation of the parameters and, conversely, every informative prior is non-informative on an appropriately transformed parameter space [38]. Even more cryptically, the induced priors on functions of parameters are generally, informative even though priors on the original parameter space are non-informative.

Another difficulty with Bayesian inference is that parameter non-identifiablity is masked in the posterior distribution. It can happen for certain situations of model and data that the likelihood surface is flat, or nearly so, in a particular region of the parameters. Thus, the data give no, or little, information on parameter values so the posterior is entirely, or mostly, dependent on the prior [38]. This is particularly a problem for complex situations where non-identifiability may not be easily recognizable [39,40].

It has been repeatedly pointed out that the posteriors of any Bayesian analysis (subjective or objective, but excluding empirical) is ineluctably a distribution of belief [16,26,41]. No amount of data changes this fact. To many, this seems a philosophical nicety that is completely overwhelmed by the ability to provide estimates for difficult problems. Latent variable and other hierarchical models can be particularly challenging to a likelihood approach because their optimization requires repeated calculation of multiple integrals of very high order [42,43]. Monte Carlo Markov chain methods allow the estimation of the posterior distributions of complicated Bayesian problems through simulation [42,43]. A great deal of milage has been made of the Walker [44] theorem, which proves that for i.i.d. data a Bayesian posterior distribution of an identifiable parameter vector *θ* converges to the sampling distribution of $\hat{\theta }$ (the maximum likelihood estimate) as sample size goes to infinity (e.g., Yamamura [45]). 

Unfortunately, real-world inference is not asymptotic. While simple models often achieve near-asymptotic behavior with quite small sample sizes, model complexities and prior infelicities can make the approach to asymptotic behavior quite slow. There is no way of knowing if a Bayesian analysis has converged to its large sample behavior except to data clone [46,47]. Data cloning is an algorithmic device to eliminate the influence of the prior from a Bayesian estimation. Data cloning provides a full range of frequentist/evidential statistical tools including maximum likelihood estimation, hypothesis testing, confidence intervals (Wald and profile), model identification via information criteria, and diagnostics for parameter identifiability [48,49,50,51]. 

A Bayesian analysis requires that the prior belief distribution integrates to 1. Thus, fundamentally, but implicitly, a Bayesian analysis assumes that the generating model is in the model set. When the generating process is not in the model set, a Bayesian analysis will be overconfident [52]. We agree with Barnard [8] (see quote above) and Box [53,54], who asserted repeatedly that “all models are wrong” and argue strongly that the generating process is almost never in any real scientific model set. Some Bayesians [55] address this problem by placing priors on models; this creates a plausible personal probability. However, this means that model probabilities are conditional on the model set. Bayesian scientists are faced with the dilemma of either claiming to be omniscient or accepting that the overarching idea of a probability for a model, that Good [35] (see quote above) felt was the common glue that held all Bayesians together, has shrunk so small that it can sleep in a teacup. All Bayesian probabilities, all Bayesian confirmations are only consistent with the real world (that is a world with model misspecification) as comparative concepts [37,56]. Some Bayesian statisticians and scientists fruitfully acknowledge these limitations and see Bayesian computation as an estimation device for complex models that needs to be followed by non-Bayesian calibration, confidence, and model-checking [57]. 

The aim of this special feature in the journal *Entropy* is to continue the development and dissemination of evidentilstatistics, building on the recent research topic in Frontiers in Ecology and Evolution (https://www.frontiersin.org/research-topics/7282/evidential-statistics-model-identification-and-science accessed 25 August 2022). We believe that evidential statistics presents an opportunity for increased clarity in scientific inference. The evidential project draws strengths from both of the opposing camps in the “Statistics Wars” while simultaneously rejecting some of the flaws of each. The evidential approach promotes efficient scientific progress by encouraging more active (yet controlled) exploration of model space [26,34]. We also believe that widespread adoption of evidential practices should act to alleviate many of the deleterious social behaviors of the scientific community that are partial causes of the replication crisis. These include “cherry picking”, “HARKing”, “the file drawer problem”, and of course misunderstanding and/or misrepresentation of the kind of uncertainty being reported [58].

We are soliciting papers that convey basic concepts and papers that convey technical subtleties sufficient to conduct real scientific research, as well as practical advice that can be incorporated into the teaching of undergraduate and graduate courses. Additionally, this special feature would benefit from application to science beyond simple examples imbedded within formulations, proofs, computer code, or logical arguments. Specifically, we are seeking papers that use the evidentialist approach to address pressing scientific questions or demonstrate the strengths and limitations of the evidentialist approach in scientific research [26,59]. 

The topic will consist of a mix of new original research, reviews, mini-reviews, opinions, and commentaries and perspectives on topics related to evidential statistics. New statistical work is encouraged, nevertheless, all papers will need to spend significant effort to explain goals, utility, and application of methods to working scientists. To further this goal, collaboration among statisticians and more empirical scientists is also encouraged. In the interest of sharpening the discussion, papers by Bayesians, likelihoodists, NP testers, severe testers, and others that explicitly critique the modern evidential project and any or all points touched on above are also welcome.
